# The Acquisition of Clause Chaining in Nungon

**DOI:** 10.3389/fpsyg.2020.01456

**Published:** 2020-07-07

**Authors:** Hannah S. Sarvasy

**Affiliations:** The MARCS Institute for Brain, Behaviour and Development, Western Sydney University, Sydney, NSW, Australia

**Keywords:** Nungon, Papuan, acquisition, clause chain, complex syntax, under-described language

## Abstract

The clause chain is an under-investigated complex sentence type, found in hundreds of languages. In a clause chain, as many as 20 or more ‘medial’ clauses with under-specified verbal predicates combine with a single ‘final’ clause with fully-specified verbal predicate. Clause chains are of interest for three main reasons: (a) the special syntactic relationship between clauses, which is neither textbook subordination nor coordination; (b) the potential extreme length of a single chain; and (c) switch-reference marking in clause chains of some languages could require speakers to plan at least one clause ahead as they speak. Research on child production of complex sentences has largely overlooked clause chains. Longitudinal data for three children aged 1;1 to 3;3 acquiring the Papuan language Nungon show that Nungon-speaking children begin producing clause chains around the age of 2;4, with a marked increase in rate of use around age 2;11. Chain length is limited to two clauses until age 3;1. Different-subject marking in medial clauses is used by all three children early, but is first attested in one-clause, ‘root medial’ contexts, rather than in chains. Bayesian statistical models confirm the strong tendency for children to use root medials in expressions of desires and commands. Children’s production of three types of complex sentences—clause chains, subordinated final clauses, and coordinated final clauses—is shown to be subject to the same type of developmental constraint; but once development reaches an adequate level for increased complex sentence production, children acquiring Nungon produce many more clause chains than complex sentences involving subordinated or coordinated final clauses.

## Introduction

This paper investigates child acquisition of the special type of complex sentence known as ‘clause chains’ in the Papuan language Nungon. Acquisition of clause chains is compared with that of subordinate clauses and coordinate clauses, and usage patterns are compared with those in child-directed speech. This section introduces clause chains in Nungon and the motivation for targeting their acquisition by children.

The clause chain is an under-investigated sentence type found in hundreds of languages around the world: from Ethiopia to Turkey to the Caucasus, the Amazon, and Papua New Guinea (Longacre, 1985, 2007; Dooley, 2010, *inter alia*). A clause chain is a sequence of one to twenty or more ‘medial’ clauses (with ‘medial’ verbs that are unmarked for tense, mood, and, often, subject), followed or preceded by a single ‘final’ clause (with a ‘final’ verb that gives all tense, mood, and subject information). Clause chains’ most basic function is the description of sequentially related events, actions and states. An eight-clause chain from the Papuan language Nungon is shown in (1), from an adult recollection of preparations for a hunting trip. Throughout this paper, medial clauses are in single curly brackets, and final clauses are in double curly brackets, following the convention in Sarvasy (2017a).


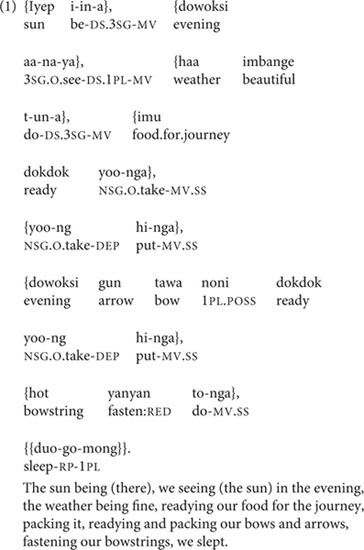


Co-referentiality of subjects across clauses in the chain is obligatorily indicated on each medial verb through switch-reference marking (Haiman and Munro, 1983; van Gijn and Hammond, 2016). When producing the medial verb of a medial clause A, a Nungon speaker signals in advance whether the subject of the next, as-yet-unspoken, clause B will be exactly co-referential with the subject of clause A or not. If the subject will be co-referential, this is a ‘same-subject’ (SS) context, and the medial verb of clause A does not inflect to index the subject of clause A. But if the subject of clause B will not be exactly co-referential with that of clause A, then this is a ‘different-subject’ (DS) context, and the medial verb in clause A must inflect to index the person/number of the subject in clause A (No information is given in advance about the person/number of the subject in clause B; it is simply understood that clause B’s subject argument will differ from the subject of clause A).

The first three medial clauses of (1) are marked for DS; their subjects are, in order: ‘the sun,’ ‘we,’ and ‘the weather.’ (Note that subject characteristics like animacy do not affect switch-reference marking, which strictly follows grammatical subjecthood.) The remaining four medial clauses are marked for SS, but their shared subject is not made explicit until the final clause at the end of the chain, which reveals that its subject, and that of the four preceding medial clauses, is ‘we.’ As a ‘final’ clause, this clause has a ‘final’ verb: these are always inflected for tense or mood and subject person/number. In Nungon, SS medial verbs receive only an unchanging suffix *-a* (*-nga* after vowel-final verb roots), but DS medial verbs inflect to indicate their own subject’s person/number.

Speakers of clause chaining languages like Nungon have a third structural option for complex sentence formation, beyond the two available to speakers of English and other Western European languages. That is, Nungon speakers can not only produce: (a) complex sentences with up to 25 or more medial clauses and a single final clause ‘chained’ together (as in example 1), but they can also produce complex sentences more like those of English, in which: (b) a main final clause subsumes one or more subordinate final clauses, or (c) two or more final clauses are coordinated, using prosody or conjunctions. It is generally accepted that clause chaining represents a special type of ‘asymmetrical’ coordination in which the medial clauses are morpho-syntactically dependent (unlike English coordinated clauses), but not syntactically embedded (unlike English subordinate clauses; Foley and Van Valin, 1984; Nonato, 2014; Weisser, 2014).

Further, examination of a corpus of monologual Nungon adult texts belonging to narrative and other genres shows that clause chain distribution in Nungon monologual storytelling is frequent and highly predictable, with number of chains per text increasing linearly as text length increases. In other genres, such as essays written in Nungon (Sarvasy and Ögate, 2019), clause chains are shorter and sparingly used; this relates to clause chains’ primary function to describe sequential events and actions. Nungon clause chains thus differ from complex sentence structures in English, which have unpredictable distributions (Barker and Pederson, 2009).

Clause chains’ morpho-syntactic differences from subordinate or simple coordinate complex sentences, their potential extreme length (chains of over 100 clauses have been attested in some languages: Wise, 2018), and switch-reference marking, which apparently requires speakers to plan chains at least two clauses at a time, are all of relevance to the study of child acquisition of complex sentences more broadly. Sarvasy (2019b) showed that the earliest verbs produced by one child acquiring Nungon are tensed, ‘final’ verbs (although the child does go through a phase of optionally using a morphologically simplified, ‘root nominal’ form alongside the well-formed ‘final’ verbs). Medial verbs were not among the earliest forms produced. The timing and general trajectory of clause chain production in Nungon have not yet been investigated. The research questions tackled in this paper on child acquisition of Nungon clause chains are:

(a) How does the development of clause chaining in Nungon pattern relative to the development of subordinated and coordinated final clauses?
(b) Do Nungon-speaking children produce chains of three or more clauses from the beginning of complex sentence production, or are their early chains limited to two clauses?
(c) If switch-reference marking requires advanced sentence planning skills, could this mean that children’s clause chain production necessarily lags behind their production of subordinated or coordinated final clauses (which are not marked for subject co-reference across clauses)?
(d) Do children produce SS marking before DS marking, which entails conception of sentences involving more than one subject?

The following section gives basic background on the Nungon language and compares Nungon clause chains to other complex sentence types.

## Nungon Clause Chains: Background

Nungon (Sarvasy, 2013, Sarvasy, 2014a,b, 2015a,b, 2016, 2017a,b,c, 2018b, 2019a,b; Sarvasy and Ögate, 2019; Sarvasy et al., 2020) is a Papuan language of the Finisterre-Huon language family, spoken by about 1,000 people in the highest inhabited reaches of the Uruwa River valley, Morobe Province, Papua New Guinea. Nungon epitomizes the unmatched linguistic diversity of Papua New Guinea (PNG)—and the difficulties inherent in drawing linguistic lines—in that ‘Nungon’ is actually an umbrella term for four different dialects, belonging to the villages Kotet, Towet, Yawan, and Worin, which differ in roughly 10–20% of lexicon and in portions of their phonetic and phonological systems. Astoundingly, these different dialects are spoken in villages located maximally 30–45 min walking from each other. The four Nungon dialects further belong to a larger, oval-shaped dialect continuum within the Uruwa River valley, in which every village community historically had its own distinct dialect (Sarvasy, 2013).

Full descriptions of the Nungon verb and inflection categories are in Sarvasy (2014a, 2017a). As noted in the Introduction, the term ‘final’ here refers to a category of verbal inflection corresponding to the inflections possible on the verb of the final clause of a clause chain. Nungon final verbs occur in the last clause of clause chains and are also the verb forms used in non-chained sentences. Final verbs are obligatorily marked for mood (Immediate or Delayed Imperative), tense (Remote Past, Near Past, Present, Near Future, or Remote Future), or reality status (Counterfactual), and always index subject person/number (with three numbers distinguished: singular, dual, and plural).

Subordination in Nungon involves a main final clause subsuming one or more subordinated final clauses marked with the clitic = *ma.* The subordinated clause can either function as an argument of the main clause, as in (2), or be connected to the main clause with looser semantics, as in (3).


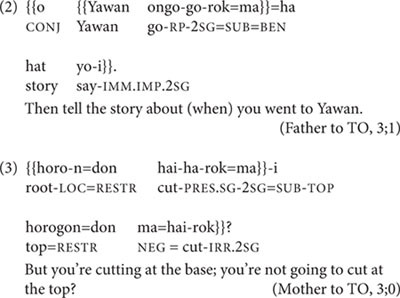


Coordination of final clauses in Nungon involves either simple juxtaposition of final clauses, with only prosodic linking (as in example 4), or coordination of final clauses through the use of conjunctions such as ‘that is,’ ‘but,’ ‘however,’ or the adverb *huttai* ‘actually,’ which is used in conditional statements, as in (5).


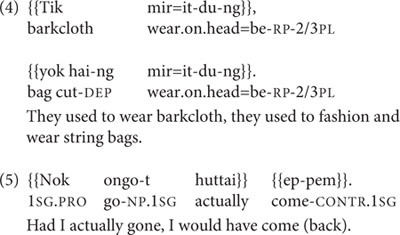


Both final clause subordination and coordination are markedly rarer than clause chaining in a sample of 49 adult Nungon narratives from 18 speakers (Impressionistically, final clause coordination is also rarer in that genre than final clause subordination).

Nungon clause chains were exemplified in (1). The primary and most lexically flexible function of Nungon clause chains is to describe a sequence of related events, actions, or states, as in (1). Within this function, events in a chain are generally understood to be consecutive, and presented in the temporal order in which they occurred/will occur (Farr, 1999), though there is the possibility that the actions or states described in some pairs of clauses within a chain can be understood as overlapping in time. Chains such as that in (1) have no upper length limit, and there are no restrictions on the types of verbs that can occur in them, nor on their positions in the chain.

Among clause chains that describe sequential events and actions, certain lexical pairings within the chain can be considered semi-conventionalized. For instance, Nungon has several individual verbs to express the bearing of non-human animal(s) or object(s) from one place to another: *ke-/he-* ‘bring it/them,’ *ku-/hu-* ‘take it/them away,’ *köö-/höö-* ‘raise it/them,’ *koo-/hoo-* ‘lower it/them.’ Use of these verbs glosses over the component actions involved in such carrying or moving. An alternative is to express a similar meaning using an SS two-clause chain with the verb *to-/yoo-* ‘take up it/them’ in the first clause, followed by a verb of motion, such as *ongo-* ‘go,’ *e-* ‘come,’ *öö*- ‘ascend,’ or *oo*- ‘descend,’ in the second clause. The sequencing of verbs in such a chain, with ‘taking it up’ always preceding ‘going’ or the other motion verbs, makes logical sense: the object cannot be carried to another place in the course of the ‘going’ unless it has already been taken up by the goer. While the pairing of ‘take up it/them’ with a motion verb can be considered somewhat conventional, it still allows for ample speaker flexibility: not only does the speaker have a choice of motion verbs, but the speaker can also insert other linguistic material between ‘take up it/them’ and the verb of motion, as in (6), from an adult personal narrative.


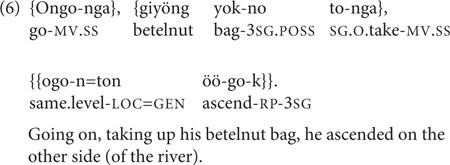


There are also two Nungon constructions in which SS two-clause chains have grammaticalized, and could be said to function largely as monoclausal constructions. The Nungon Continuous aspect construction comprises any lexical verb in SS medial form immediately followed by *it-* ‘be,’ without any intervening material, and usually, without a pause. This is the usual way in the language to describe an in-progress event, action or state, illustrated in (7). Here, the single clausal ‘was beating out song’ is the more felicitous interpretation, rather than the two-clause ‘beating out song, was/stayed.’ Despite this, there are indications from both adult and child speakers that the two verbs of the Continuous aspect construction can be optionally expanded into a clearly biclausal chain, either by inserting additional linguistic material between the first medial verb and *it-* ‘be,’ as in (8), or by adding a pause between them.


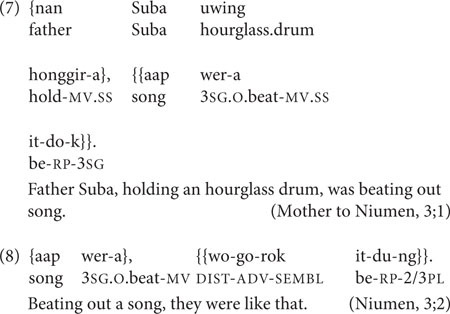


The other grammaticalized two-clause chain type is called the Inferred Imperfective aspect construction. Here, a special form of the verb *to-* ‘do’ indicates a degree of non-firsthand information source, or evidentiality (Sarvasy, 2018a). This can be immediately preceded by a medial verb in what is formally a SS two-clause chain, as in (9), where the child Niumen’s insect bites are visible to his mother, but the insects who bit him are unknown.


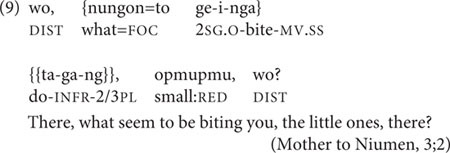


In the child–parent interactions used here, the Inferred Imperfective sometimes occurs with its extended function: to express incredulous annoyance, as in Niumen’s complaint to his mother in (10).


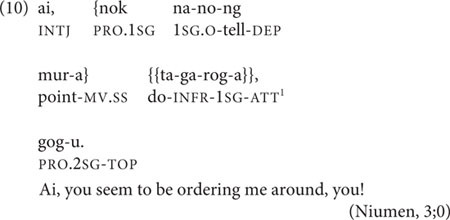


The Inferred Imperfective construction differs from the Continuous aspect construction in that the special form of ‘do’ obligatorily occurs in a final verb form, while the verb ‘be’ in the Continuous aspect construction can itself take a medial form within a clause chain.

Both aspectual constructions are included in the present study. They are counted as two-clause chains, though given special codes; for some analyses, they are omitted or kept separate from other clause chains. There are indications in Niumen’s transcripts at 2;11 and older that the child already understands that these constructions are formally composed of two separate words, if not clauses: Niumen is able to omit the verb *it-* ‘be’ elliptically in response to a question framed in the same aspect and to insert a demonstrative between the medial verb and *it-* ‘be’ in (8).

Though Nungon medial clauses are morphologically dependent and canonically occur within clause chains, they can occur independently, outside clause chains, in both child and adult speech, under certain pragmatic conditions (Sarvasy, 2015a). Such medial clauses are called ‘root medials’ here, in a nod to ‘root infinitives’ of child German and other languages (Rizzi, 1994; Sarvasy, 2019b). In Nungon, some of the basic pragmatic functions of root medials are: (a) to command, with slightly different force or politeness than dedicated imperative forms, (b) to elaborate on a preceding clause chain, and (c) to express something elliptically, or with a ‘trailing-off’ effect. An example of a root medial used to command is in (11).


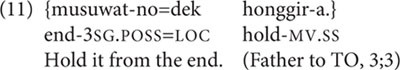


## Materials and Methods

The present dataset comes from a larger longitudinal study of five children acquiring the Towet village dialect of Nungon in a village setting; more information on the larger study is on its CHILDES page, https://childes.talkbank.org/access/Other/Nungon/Sarvasy.html (MacWhinney, 2000). The five children were aged 1;1, 2;1, 2;10, 3;5, and 3;8 at the study’s outset. All were recorded interacting with one or more parents and other family members for 1 h monthly over a 25-month period, except the youngest, Abraham, who entered the study later than the others, and was recorded throughout a 19-month period, through age 2;7. Of these children, transcripts from the youngest three are included in the present study. These were the ones that were fully coded and available for analysis. The age range from approximately 2;0 to 3;3 is also especially useful for examination of the early development of clause chaining, since the two older children already seem to have adept command of clause chains of varying lengths from their earliest recording sessions (at 3;5 and 3;8): this was part of the reason that the three youngest children’s transcripts were coded first. Transcription and coding of an additional 60 h from a denser corpus with three other children, aged 2;1 to 2;7 initially and tracked intensively for 5 months, is now underway.

Recording sessions took place in or around the children’s homes with participants seated on the floor or ground. At the beginning of each session, the interviewer (a local Nungon speaker and classificatory kin of all children) positioned a small tripod-mounted Zoom H5 audio-recorder on the floor or ground, with the two built-in microphones pointing toward the child. This was set to record at a 44.1 kHz sampling rate and into WAV format. A second interviewer held a Canon digital camera recording in VGA video for the duration of most sessions. After the interviewer turned on the audio recorder and pressed ‘record,’ s/he sometimes left the scene, leaving parent and child alone to converse (or accompanied by the video-recording interviewer). After approximately 1 h, the interviewer returned and ended the session. The parents understood that the goal of the sessions was to track the children’s linguistic development, and that they were meant to solicit speech from them. In some sessions, the parents relied on props as conversational prompts, especially printed images, including two picture-books left by the researcher: a compilation of photos of Towet community members, and *Koko’s Kitten* (Patterson and Cohn, 1985). The parents also often prompted the children to talk about recent excursions to mountain farm plots, the high forest, or the coast, recount recent village events, and comment on or call out to people passing by the scene of the recording. In two sessions (at 3;0 and 3;3), Towet Oe (TO) and her father chat as the child prepares a meal and her father serves as her assistant. TO and Abraham’s fathers were present in some of the sessions, and in one to two transcripts, served as main interlocutor in the absence of the child’s mother, but Niumen’s mother is his primary interlocutor in all the transcripts used here.

The recording sessions were digitally transcribed by the four interviewers on Lenovo 10.1” laptops in the villages in Wordpad using mid-CHAT format (MacWhinney and Snow, 1985, 1990), or, for sessions after September 2016, directly into CLAN. Of these transcriptions, ten (the five first made available on CHILDES, plus the first four for TO) have been thoroughly checked by the researcher against the audio recordings. Although two of the transcribers used more orthographic idiosyncrasies (such as separating the final syllable of polysyllabic words from the rest of the word with a space) than the others, these transcriptions were found to be highly faithful to the recordings.

For the present study, only the first 11 transcripts for Abraham (1;1 through 2;1) and the transcript from 2;4 were available. The first 15 transcripts for TO were available (2;1 through 3;3), and the first six transcripts for Niumen were used (2;10 through 3;3). These were coded in different ways.

In the first transcript for Niumen, and all 15 transcripts for TO, all child verb forms were coded by hand into a text file version of the transcript. Verbs were coded as to inflection and subject person/number. Medial verbs were coded as such, and received additional codes if they were marked for DS, occurred within a Continuous aspect or Inferred Imperfective construction, or were root medials. In the TO transcripts, all parent verb forms were similarly coded as well, but in the Niumen transcript at 2;10, only parental medial verbs were coded. The remaining five transcripts for Niumen had been fully glossed in CLAN for inclusion in the CHILDES database, so no further coding was done to them for the present study. Time constraints mean that parent verbs could not be coded in the transcripts for Abraham, but the child’s verbs were coded in a spreadsheet of all his utterances in the collected transcripts, obtained through CLAN.

The transcripts that had been coded by hand into a text file were then searched individually using the search function in Microsoft Word to yield individual medial verb tokens for the children, and total counts of target phenomena for children and adults. The other five transcripts for Niumen were searched for the relevant morphological glosses in CLAN. From the spreadsheet with all utterances for Abraham, coded utterances including medial verbs were culled by hand.

All 785 child utterances including medial verbs for the three children were then compiled into a spreadsheet that served as the basis for further qualitative and statistical analysis of child medial verb and clause chain production. This spreadsheet also included counts for each utterance for number of medial verbs in the chain, length (in clauses), if the chain indicated Continuous aspect or Inferred Imperfective aspect, whether the utterance included a ‘root medial’ clause or was otherwise elliptical (lacked a final clause), and four pertinent contextual codes: desire, free narrative, picture-book, and repetition. The ‘desire’ code indicated whether the utterance expressed a command or desire; this was determined by conversational context for root medials, and, for clause chains, by whether the final verb in the chain was in an imperative inflection. The ‘free narrative’ code indicated whether the utterance occurred as a child’s original narration of some past or future events, and the ‘picture-book’ code indicated whether the utterance was produced while the child and parent were engaged in describing printed images. The ‘repetition’ code indicated whether the child’s production was an immediate repetition of a parental prompt. Prompted medial verbs were not excluded from initial counts, but self-repetitions within the same utterance or immediately after were excluded. In the example utterances in the Results section, child productions that immediately follow parental prompts are distinguished from those that do not follow parental prompts; total number of prompted medial verbs per transcript per child are given in Table 1, below.

**TABLE 1:** Counts of clause chain-related verb forms produced by target children.

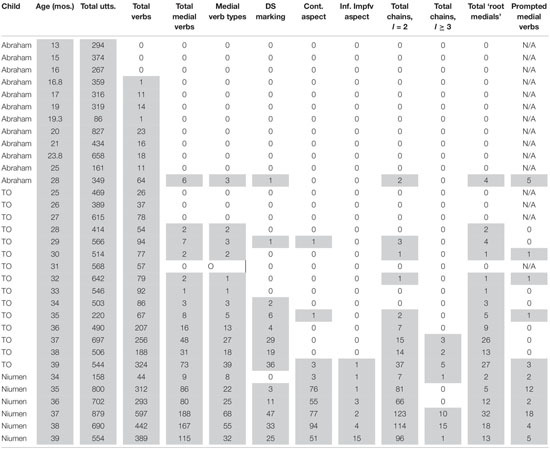

Subordinated and coordinated final clauses in the children’s speech were then culled by hand from all transcripts for the three children, yielding an additional spreadsheet with 243 utterances including these constructions. Each construction was coded according to several sub-types of coordinated and subordinated clauses, and also coded as to whether a clause was formally marked as subordinate with the marker = *ma* but occurred independently of a main clause—the subordinated equivalent to ‘root medial’ clauses. Time constraints meant that the same constructions were not also culled for parental speech.

Analysis and visualization were performed in R, using the brms, ggplot2, and tidyverse packages.

## Results

Discussion of the verb/utterance ratio as a measure for children’s verbal development begins this section, followed by summaries of overall frequencies of medial verb tokens, and of medial verbs marked for DS, across speakers in the dataset. Children’s clause chain productions are then examined in depth, followed by a brief discussion of clause chaining in child-directed speech, then statistical model results.

### General Verbal Development

In the statistical models later in the paper, both age and a proxy for verbal sophistication, verbs/utterance, are used as independent variables. This section introduces the verbs/utterance ratio.

The ratio of verb tokens to total utterances within a single transcript is taken as somewhat indicative of verbal sophistication, where utterance is equated with one time-marked entry in the CHILDES/CHAT format (MacWhinney and Snow, 1985, 1990). Nungon does allow non-elliptical verb-less clauses (Sarvasy, 2017a), especially to express identity and equation (‘X = Y’), where English speakers would use the copula *be.* In the present study, verb-less clauses are most often present in question-answer sequences about the identities of people in printed material [*ngo numa?* ‘this (is) who?’ *wo nan* ‘that (is) Father’], or the owners of items at hand [*ngo numa* = *hon?* ‘this (is) whose?’ *wo nagain* ‘that (is) mine’]. The ratio of verb-less clauses to clauses with verbal predicates are expected to vary from transcript to transcript, depending on discourse content and context. In fact, however, when instances of maternal *ngo numa?* ‘this (is) who?’ and *ngo nungon?* ‘this (is) what?’ were tallied for Niumen’s and TO’s mothers, these were found to occur in between 12.43% and 16.57% (mean: 13.33%) of all Niumen’s mother’s utterances, and 0.00% and 7.71% (mean: 2.29%) of TO’s mother’s utterances. This shows that the amount of variation across transcripts in use of these by a single speaker is relatively small.

Another possible confounding factor for use of the verb/utterance ratio as an indication of verbal sophistication is the possibility that it could mask discourse-related asymmetries between child and caregiver speech in these dialogic recording sessions, for instance, if children tend to give elliptical, verb-less answers to questions originally posed with verbs (anticipated for the measure of MLU by Johnston, 2001). Impressionistically, this is not overwhelmingly the case for Nungon-speaking children, and Nungon’s status as a pro-drop language with a strong preference for argument omission might play a role in dispreferencing verb-less answers to questions that include verbs.

**FIGURE 1:**
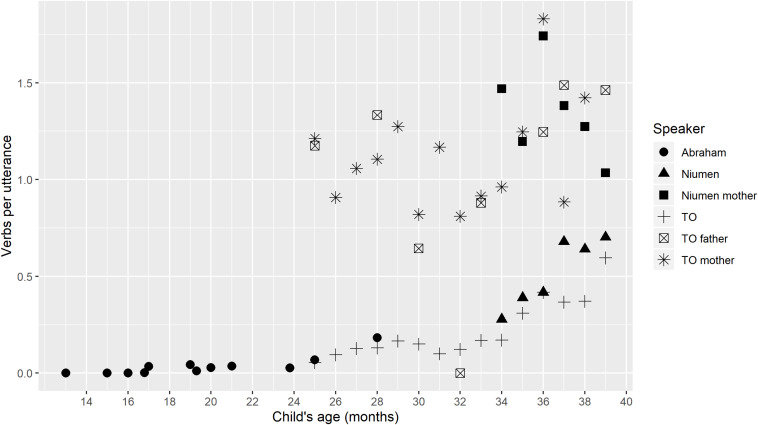
Verbs per utterance by child’s age.

Indeed, the ratio of verb tokens to total utterances does increase with age for the children, and all three appear to follow similar developmental trajectories when this measure is applied. Figure 1 shows stable, low rates of verb production from about 16–24 months (Abraham), then gradual increases from about 24–33 months (Abraham and TO), and finally, accelerating rates of increase from about age 34 months on (TO and Niumen). The ratios for different children at the same ages are almost all within 0.1 of each other. This is reassuring: despite this study’s limitations in using only three children studied for different lengths of time and age ranges, the children appear to follow similar developmental trajectories.

The youngest child, Abraham, produces no verb tokens for the first three recording sessions (ages 13–16 months). From 17 to nearly 24 months, his ratios are all between 0.0 and 0.04. But his ratio at age 25 months is 0.068, representing a minor jump to a ratio which is similar to, though slightly higher than, TO’s verb/utterance ratio at the same age: 0.055. At 28 months, Abraham’s verb/utterance ratio is higher still, at 0.18; this is slightly more than, but in a similar range to, TO’s ratio at the same age: 0.13. Thus, Abraham’s net increase in verb/utterance ratio between ages 25 and 28 months is 0.037/month, or almost the same increase each month in his 26th, 27th, and 28th months as his total net gain between 16 and 24 months. TO starts at a more advanced stage than Abraham, but her development over the study period can also be divided visually into two stages: a slower increase between 25 and 34 months, then more rapid increases between 35 and 39 months. Niumen’s verb/utterance ratio increases between the ages of 34 and 39 months are at a similar scale to those of TO.

All parental verb/utterance ratios are generally higher than those of the children, with the exception of one data point from TO’s father, where he produced 104 utterances, including 67 verbs; that ratio (0.64) is the same as the child Niumen’s ratio at age 38 months (442 verbs in 690 utterances). Niumen’s verb/utterance ratios at four of the six ages are higher than TO’s at the same ages, but the difference between the two children at any one point is always less than the difference between the higher child’s ratio and the lowest of the parents’ ratios at that point. TO’s mother’s verb/utterance ratios are within the range of 0.8–1.9, while Niumen’s mother’s ratios for the smaller age range in which data is available are 1.2–1.8. Indeed, mother’s verb/utterance ratio is predictive of child’s verb/utterance ratio, as will be seen in the statistical model results.

### Medial Verb Development

A first clue to clause chain use by all speakers is their use of medial verbs, which only occur in medial clauses: either medial clauses within clause chains, or ‘root medial’ clauses (pragmatically-driven independent uses of medial clauses, outside of clause chains).

**FIGURE 2:**
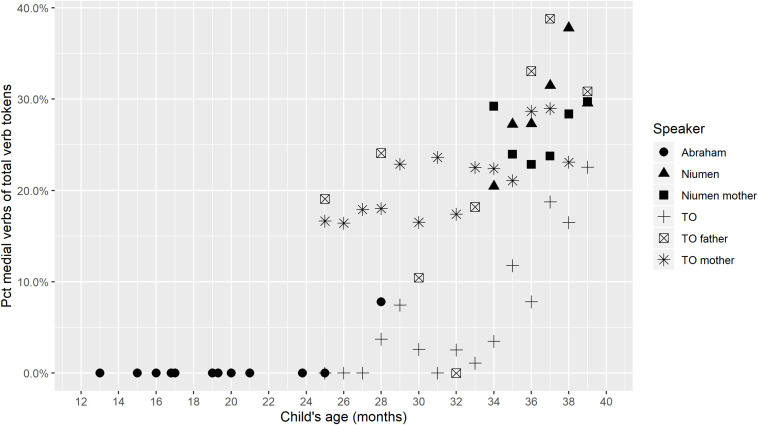
Medial verb tokens per total verb tokens.

Medial verb use can be quantified through the percentage that medial verb tokens comprise of the total verb tokens produced by a speaker in a single transcript, in Figure 2. If the percentage of medial verbs is higher for a speaker in transcript A than in transcript B, this indicates that: (a) the speaker produced more clause chains or root medials in A than in B, *or*: (b) the speaker produced the same or fewer clause chains in A than in B, but chains in A tended to be longer (comprising more clauses) than chains in B, *or*: (c) the speaker produced more, and longer, clause chains in A than in B.

A few general trends are evident in Figure 2. Throughout the study period, TO’s parents have higher percentages of medial verbs than TO in a given transcript, but TO’s percentages from 37 to 39 months fall in the same range as her parents’ did in the period up to 33 months. This means that the parents could be adjusting the complexity of their clause chaining based on TO’s own verbal sophistication (Bohannon and Marquis, 1977; Soderstrom, 2007, *inter alia*). In only one instance, from the last transcript for Niumen at age 39 months, does a child’s percentage of medial verbs surpass that of a parent in the same transcript.

**FIGURE 3:**
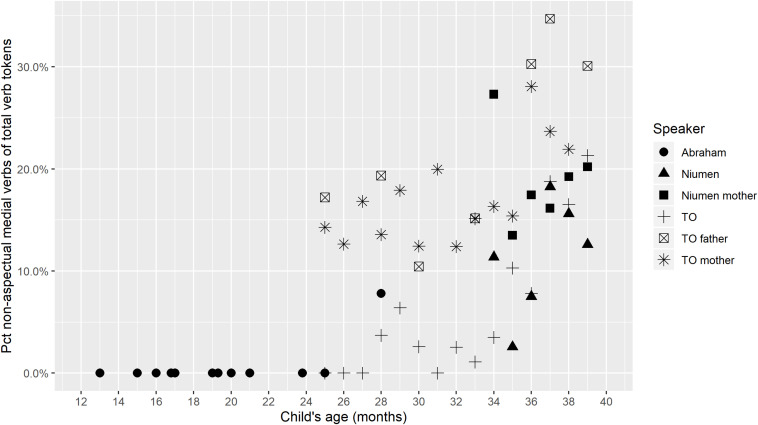
Medial verb tokens not in aspectual constructions, per all verb tokens.

Although TO and Niumen’s verb/utterance ratios are similar at the same ages (Figure 1), Niumen produces higher percentages of medial verbs (of all verb tokens) than TO from 34 to 39 months. This difference is largely due to Niumen’s more frequent use of the Continuous aspect construction. Figure 3 shows that if aspectual two-clause chains are omitted from counts, Niumen and TO’s proportional medial verb usages are similar in the period from 34 through 39 months. Of the 359 instances of the Continuous aspect construction produced by TO and Niumen during the study period, 337 (or 93.9%) occurred in the context of picture-book or other printed image description. Picture-book description was a frequent activity for Niumen and his mother in the recording sessions, but not as much for TO and her parents.

Overall, Figures 2, 3 show that children’s medial verb production increases with age. It also appears that parental fine-tuning may be occurring, with TO’s mother producing on average 5.7% more medial verbs in the period of 34–39 months than in the first nine transcripts, and TO’s father producing on average 6.0% more medial verbs from 34 to 39 months than from 25 to 33 months.

**FIGURE 4:**
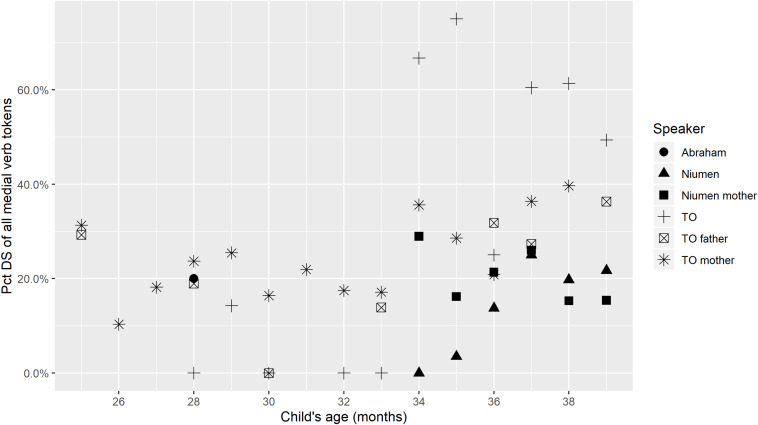
Percentage of medial verb tokens with Different-Subject marking.

**FIGURE 5:**
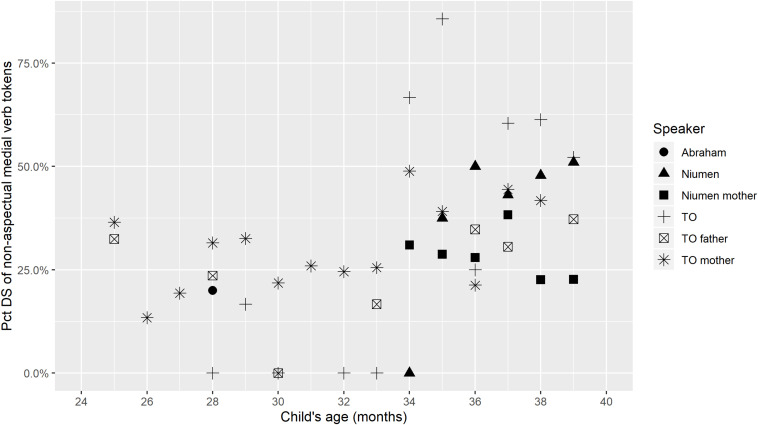
Percentage of non-aspectual medial verb tokens with Different-Subject marking.

The obligatory switch-reference marking on Nungon medial verbs means that a further division can be made among medial verbs, even once those occurring in aspectual constructions are optionally filtered out. Our research questions hinted at the possibility that children might produce SS and DS medial verbs with different frequencies and at different developmental stages. In the last global proportion counts in this section, we examine proportions of DS-marked medial verbs across speakers. Figure 4 shows the percentage of medial verb tokens that are marked for DS in a given transcript, by speaker, and Figure 5 gives the same percentage for only non-aspectual medial verbs.

Figures 4, 5 imply a limited relationship between age and use of DS marking: there seems to be reduced incidence of DS marking in the early stages of medial verb production (approximately 2;4 through 2;9), but once medial verb use increases, around 2;10–2;11, DS marking is robustly present on 33.3–51.0% of Niumen’s non-aspectual medial verbs, and 25.0–85.7% of TO’s non-aspectual medial verbs. Niumen’s use of DS marking between 2;11 and 3;3 could show a slight upward trend, but TO’s use in the same age range does not show any indication of this. In the very first transcripts with medial verb productions for TO and Niumen, all their medial verbs happen to be SS, but these transcripts stem from recording sessions at very different ages: 2;4 for TO and 2;10 for Niumen. Both children then produce a mix of SS and DS forms in the very next recording session. Abraham’s first six medial verbs, at 2;4, include one DS-marked medial verb in a root medial clause, but this is one of his utterances that follows a parental prompt.

TO’s proportional use of DS is in contrast to her parents, whose percentages of DS-marked medial verbs show a potential, slight increase from 34 months, but whose percentages still remain between 10 and 40% for the entire study period. Attributing the parents’ increase in DS marking to fine-tuning may be untenable, since switch-reference marking depends on subject maintenance or changes within clause chains, which relates to the meaning of each chain. However, it could be the case that the parents begin to describe more semantically complex situations in their child-directed clause chains as the child’s perceived verbal sophistication increases.

We now turn to detailed analysis of children’s clause chain production in Nungon.

### Clause Chain Production by Children

Overall results for the target children are in Table 1. Table 1 gives, for each child at each age: total utterances, number of verb tokens, number of medial verb tokens, number of medial verb types, number of DS-marked medial verbs, number of Continuous aspect constructions, number of Inferred Imperfective aspect constructions, number of clause chains containing two clauses, number of clause chains containing three or more clauses, number of root medials (independent medial clauses), and number of prompted medial verbs.

In Table 1, medial verb types can be taken as an estimate of the lexical range of medial verbs. It should be noted that this measure does not take the lexical verb of the following clause into consideration. This means that all tokens of the SS medial verb *to-nga* ‘taking it up’ illustrated in example (6) would be classed as the same ‘medial verb type’ regardless of the particular verb of motion used in the following clause of a two-clause chain. Two medial verbs of the same type do not necessarily occur in identical clause chains—in fact, in many cases, they do not. SS-marked and DS-marked medial verbs were classed as different types, such that the SS verb *ongo-nga* ‘going’ would be a different ‘medial verb type’ than the DS, 2sg medial verb *ong-i-ya* ‘you having gone,’ despite the fact that they are formed from the same verb root.

As noted before, Abraham produces no recognizable medial verb tokens between ages 1;6 and 2;1. There is a gap in data availability for him for 2;2 and 2;3; in the last available transcript, at 2;4, he produces six medial verbs, of which five occur in prompted utterances (either root medials or two-clause chains). The detailed examination here will thus focus on TO’s and Niumen’s productions.

Figure 2 indicated that medial verbs begin to form greater percentages of total verb tokens from about age 2;10 (34 months). This period also marks the beginning of a marked increase in medial verbs produced in bona fide clause chains, as opposed to root medial clauses. Table 1 shows that TO produces independent root medial clauses more consistently than two-clause chains through age 2;10 (34 months). She continues producing equal or greater numbers of root medials than standard medial + final two-clause chains through 3;1 (37 months), but in the last 2 months of the study period (3;2 and 3;3, or 38 and 39 months), medial verbs in clause chains outnumber medial verbs in root medial clauses. The shift in balance is not due to any decrease in TO’s root medial token production, but rather stems from TO’s increased production of bona fide clause chains.

As noted in the discussion of Figures 4, 5, TO’s first three two-clause chains, produced without prompting at 2;5 (29 months), are all SS. But even though her first production of a DS-marked medial verb occurs in that month’s transcript, that token occurs in a root medial clause, not a true two-clause chain. TO’s next two productions of DS medial verbs occur at 2;10: these are, again, in root medial clauses. It is not until 2;11 that TO produces her first two-clause chains with DS marking. So although the DS form is used early in root medial clauses, there is no positive evidence from TO that the child can produce DS-marked medial verbs in actual clause chains before 2;11 (It is hoped that the new, denser corpus targeting verbal development in three additional Nungon-speaking children between 2;0 and 3;0 will help establish whether this is also the case for children recorded for 4 h monthly instead of one).

We now examine some of the actual child productions in the dataset. Since Abraham’s only clause chains are almost all repetitions of parental prompts, the detailed discussion here focuses on development by TO and Niumen. At 2;4, TO produces her first two medial verbs in the transcripts. Both of these are un-prompted by her parents. The first occurs in the root medial utterance in (12).


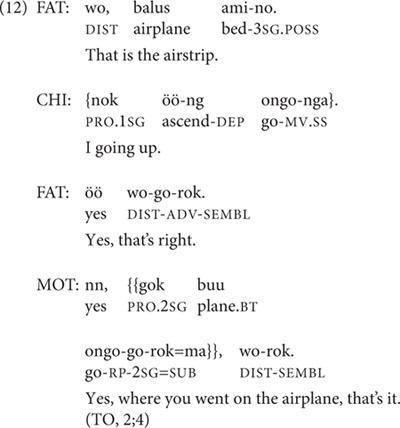


TO’s other medial verb token at 2;4 occurs in a two-clause utterance with the medial and final clauses’ ordering reversed, seen in (13). This non-canonical ordering is also attested in the speech of adults (Sarvasy, 2015a), when an elaboration or explanation is supplied after the speaker has already concluded a preceding clause chain by uttering a final clause.


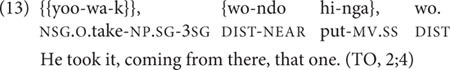


This follows her mother’s pointing out an image in a picture-book (*Koko’s Kitten*); it is unclear what TO means by it. The medial clause here was coded as a root medial clause, since it lacks a following final clause.

At 2;5, TO produces seven utterances including medial verbs, of which four are root medial clauses, one is a Continuous aspect two-clause chain, and two are SS two-clause chains that refer to distinct actions. None of these follow parental prompts. One of the root medial clauses has a medial verb that is inflected for DS, but all other medial verbs are SS. The Continuous aspect chain is shown in (14), and the two SS two-clause chains are in (15) and (16).


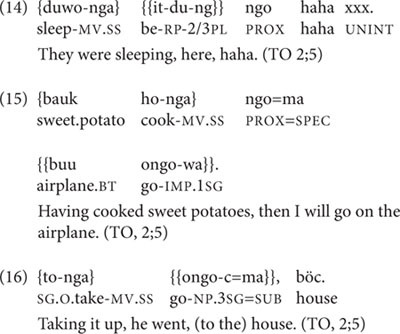


The chains in (15) and (16) differ slightly in the degree of close association between the actions of the first and second clauses. In (15), the cooking of sweet potatoes and getting on an airplane are separated in time and space; nor is there any conventionalization about this combination. In contrast, the SS clause chain in (16) fits one conventional way of carrying something from one place to another, as illustrated in (6), above, and the ‘taking up’ is immediately followed by the ‘going.’

The second verb in (16) is ambiguous between a ‘root nominal,’ the nominalized form of the verb used as a main clause predicate in child-directed and child speech (discussed extensively in Sarvasy, 2019b), and the Near Past form of this particular verb with a 3sg subject. Unlike root medials, root nominals are not found in adult-directed adult Nungon speech. It should be noted here that there is no evidence in the dataset for any morphological reduction of medial verb forms or other un-adult-like features of child clause chain productions—save for one three-clause chain produced by TO at age 3;3 in which one of the DS-marked medial verbs should be SS-marked, given as example (25) below.

At 2;5, two of TO’s four root medial clauses express TO’s immediate desires or requests, as seen in (17), in which the medial verb bears DS marking, and (18). Another root medial clause apparently describes a picture of the kitten in the picture-book *Koko’s Kitten* and resembles a Continuous aspect construction without the verb ‘be’ (in 19), and the fourth root medial clause describes a friend’s mother’s having gone away (in 20).


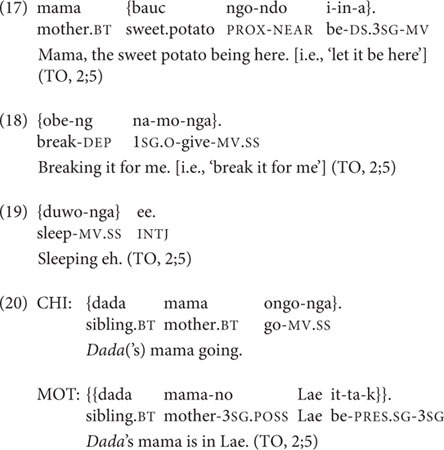


Despite the fact that TO produces three two-clause chains at 2;5, and a potential precursor (with medial and final clauses switched) at 2;4, her clause chain use does not increase in the subsequent recording sessions over the next 5 months. TO’s transcripts from 2;6 to 2;10 include her productions of six root medial clauses (of which four are SS and two are DS) and only two two-clause chains, both SS and lexically identical to each other (‘coming, see it!’ although produced in different transcripts, at 2;6 and 2;9), and both produced at the prompting of her mother to call out to a sibling, as seen in (21).


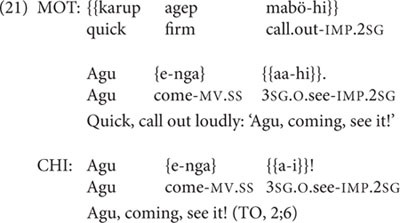


In the transcript from age 2;11, TO shows a slight increase in two-clause chains over previous months, while continuing to produce root medial clauses as well. Both two-clause chains she produces at 2;11 have DS-marked medial verbs: these are her first DS two-clause chains, since all her DS-marked medial verbs in earlier sessions occur in root medial clauses, not two-clause chains. From 2;11 through 3;1, TO’s production of two-clause chains continues to expand in both lexical and semantic versatility and quantity. For instance, the combination of verbs in the DS two-clause chain in (22) is not conventionalized and shows some linguistic creativity on the part of the child. Similarly, the postposed expansions on the two-clause chain in (23) indicate an enhanced expressive ability over TO’s earliest clause chains.


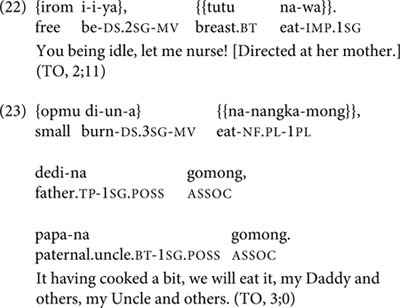


In the session at 3;1 and the two following sessions, TO shows mastery of complex, creative three-clause chains, including DS-marked as well as SS-marked medial verbs. One of these is in (24).


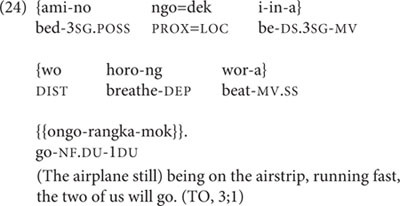


At 3;3, TO produces the only incorrect switch-reference marking on a medial verb attested for any of the children in the entire dataset. This is thus the single incorrectly switch-reference-marked token out of a total of 193 medial verb tokens produced by TO, with no incorrect switch-reference marking in any of the 6 medial verb tokens produced by Abraham, nor the 645 tokens produced by Niumen. TO’s sole medial verb token with incorrect switch-reference marking occurs within the three-clause chain in (25).


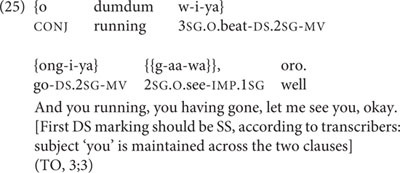


Niumen’s earliest speech sample, at 2;10, includes eight utterances with medial verbs (all SS marked, and including one root medial clause; of 158 total utterances). Two of these follow parental prompts. The six unprompted medial verbs exhibit all three major functions of clause chains: description of consecutive actions (in 26); Continuous aspect (in 27), and Inferred Imperfective aspect (in 28).


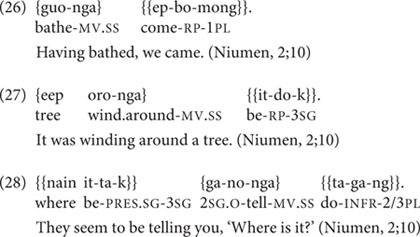


By 3;1, Niumen (like TO) produces complex, non-conventionalized chains of three or more clauses, containing multiple events and, often, DS marking (19.8–25.0% of Niumen’s medial verbs are DS marked at 3;1–3;3). But while TO never produces a clause chain of more than three clauses in the study period, Niumen’s attested unprompted chain lengths jump from only two clauses (2;10–3;0) to the range of three-to-five clauses (3;1–3;3). One of Niumen’s four-clause chains is in (29), and a three-clause chain with two DS-marked medial verbs is in (30).


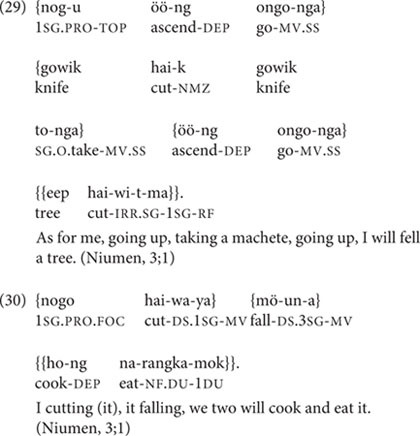


The Introduction explained that languages like Nungon have three options for complex sentence formation, compared with the two major options in English. This section concludes by comparing TO and Niumen’s non-aspectual clause chain productions with their productions of subordinated and coordinated final clauses.

**FIGURE 6:**
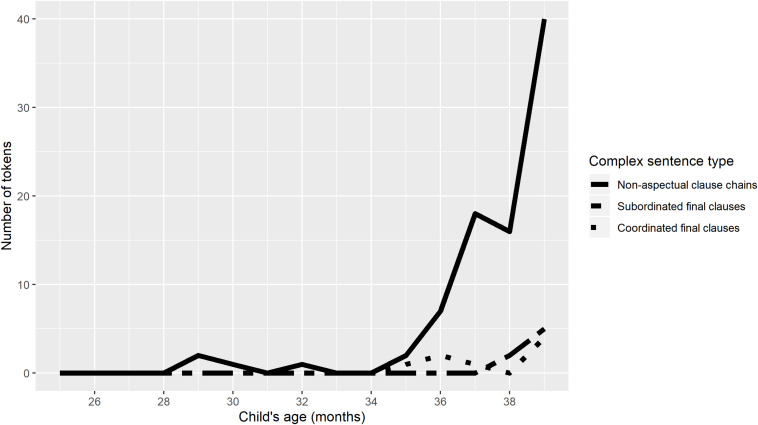
Comparison of complex sentence types, TO.

**FIGURE 7:**
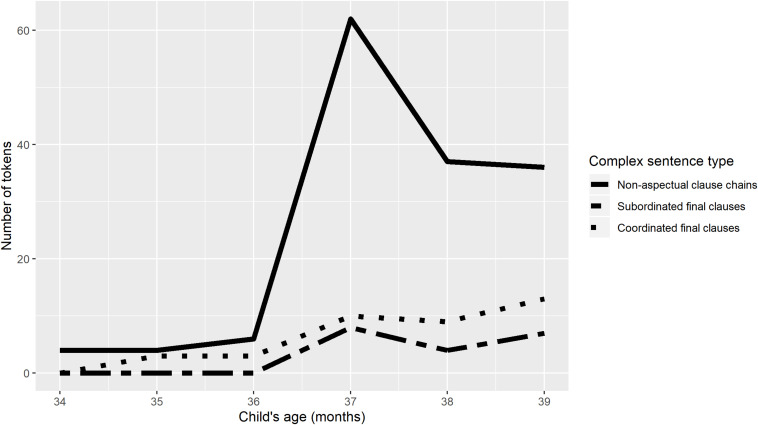
Comparison of complex sentence types, Niumen.

Figures 6, 7 show the numbers of non-aspectual clause chains of two or more clauses, complex sentences including coordinated final clauses, and complex sentences with subordinated final clauses, produced by TO and Niumen. Speech reports (‘s/he said, “I will go”’), which also superficially involve two or more final clauses, are not shown in Figures 6, 7, but speech report tokens were relatively sparse in the dataset, ranging from 0 to maximally 3 for either child in any transcript.

For both children, counts of all three types of complex sentence in Figures 6, 7 begin to increase at 2;11 or 3;0, after a sustained period of minimal complex sentence production. But for both children, the increase in complex sentence production coincides with a sharp divergence in clause chain production from the other two types of complex sentence.

Figures 6, 7 imply that children’s productions of complex sentences including two or more clauses are shaped by both cognitive development and native language characteristics. For both children, all types of complex sentence production increase around the same time, but the increase is most dramatic for clause chains, and clause chains continue to be produced in much greater numbers than subordinated or coordinated final clauses for the rest of the study period.

### Clause Chains in Child-Directed Speech

Clause chains are an integral feature of Nungon discourse, and the preferred way to describe related events or actions. It therefore makes sense that clause chains are present in child-directed speech from the earliest transcripts here, before the children themselves begin to produce clause chains. Overall results for TO’s parents and Niumen’s mother are in Table 2.

**TABLE 2:** Counts of clause chain-related verb forms produced by parents.

**Speaker**	**Child’s age (mos.)**	**Total utterances**	**Total verbs**	**Total medial verbs**	**DS medial verbs**	**Cont. aspect**	**Inf. Impfv. aspect**
TO mother	25	491	595	99	31	12	2
TO mother	26	584	530	87	9	19	1
TO mother	27	697	737	132	24	7	1
TO mother	28	487	538	97	23	24	0
TO mother	29	566	721	165	42	36	0
TO mother	30	539	442	73	12	18	0
TO mother	31	562	656	155	34	23	1
TO mother	32	568	460	80	14	23	0
TO mother	33	340	311	70	12	23	0
TO mother	34	548	527	118	42	28	4
TO mother	35	240	299	63	18	15	2
TO mother	36	183	335	96	20	1	1
TO mother	37	43	38	11	4	1	1
TO mother	38	661	940	217	86	10	1
TO mother	39	–	–	–	–	–	–
TO father	25	183	215	41	12	4	0
TO father	26	–	–	–	–	–	–
TO father	27	–	–	–	–	–	–
TO father	28	411	548	132	25	25	1
TO father	29	–	–	–	–	–	–
TO father	30	104	67	7	0	0	0
TO father	31	–	–	–	–	–	–
TO father	32	1	0	0	0	0	
TO father	33	225	198	36	5	5	1
TO father	34	–	–	–	–	–	–
TO father	35	–	–	–	–	–	–
TO father	36	374	466	154	49	12	1
TO father	37	558	830	322	88	31	3
TO father	38	–	–	–	–	–	–
TO father	39	623	911	281	102	5	2
Niumen mother	34	177	260	76	22	5	0
Niumen mother	35	990	1185	284	46	120	4
Niumen mother	36	776	1352	309	66	68	5
Niumen mother	37	901	1245	296	77	89	6
Niumen mother	38	722	920	261	40	79	5
Niumen mother	39	569	589	175	27	53	3

Conversational turns when interacting with toddlers are necessarily limited in length, but the parents here manage some respectably long and complex clause chains within their turns. Example (31), which TO’s mother directed at TO at age 3;2, is a six-clause chain within a speech report, which also includes a parenthetical two-clause Inferred Imperfective aspect chain interpolated into the main clause chain:


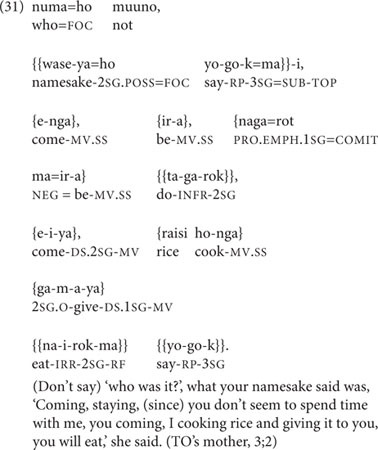


In (31), two of the five medial verbs within the main clause chain are marked for DS. Example (32) is a six-clause chain (there are eight clauses here, but the first two clauses are self-repetitions), directed from TO’s father to TO at age 3;1:


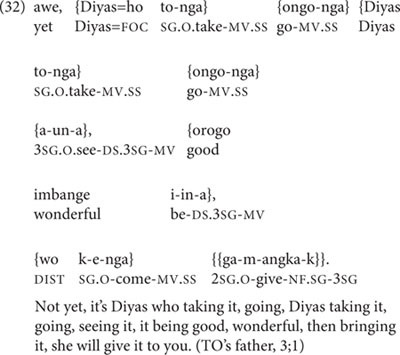


In (32), as in (31), two of the five medial verbs are marked for DS.

### Statistical Model Results

Bayesian modeling was used to test impressions from visual inspection of the data.

**FIGURE 8:**
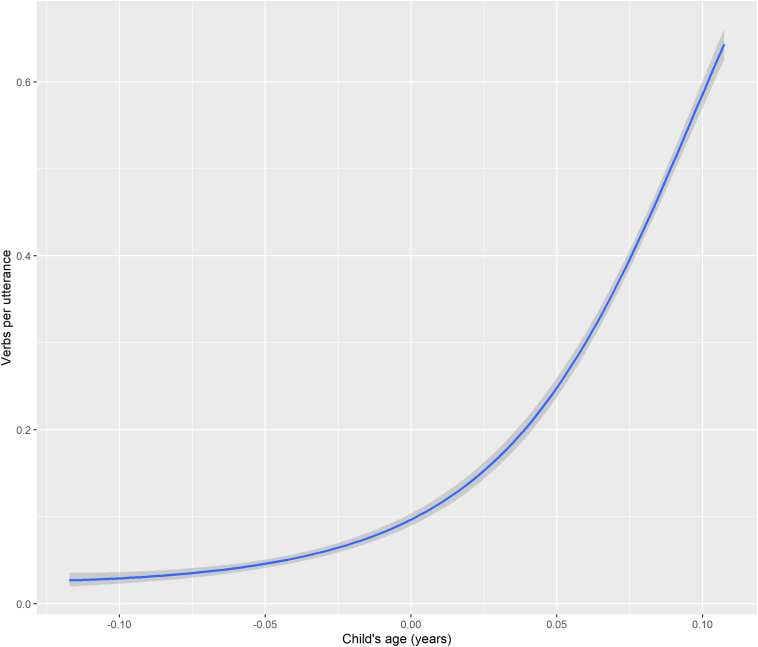
Conditional effects of child’s age and age^∧^2 on verb/utterance ratio.

First, a Bayesian logistic regression model served to test the relationship between child’s age (in years) and the verbs per utterance ratio. This showed a very strong quadratic relationship between age and the verb/utterance ratio. The conditional effects are visualized in Figure 8 and population-level effects are given as Model 1 in Table 3. The model greatly outperforms a null model using leave-one-out cross-validation.

**TABLE 3:** Population-level effects for Bayesian linear models.

**Model**			**Estimate**	**Est. error**	**1–95% CI**	**u-95% CI**	**Rhat**	**Bulk_ESS**	**Tail_ESS**
1	Age to predict verb/utterance ratio	Intercept	–1.85	0.08	–2.03	–1.71	1.00	2233	2502
		Child’s age	2.87	2.78	–0.63	9.86	1.00	1812	1977
		(Child’s age)^∧^2	198.38	23.16	141.12	230.78	1.00	1783	1998
2	Age and verb/utterance ratio to predict medial verbs/verbs ratio	Intercept	–2.68	0.15	–2.98	–2.37	1.00	2753	3198
		Child’s age	–1.13	1.77	–5.73	1.38	1.00	3050	1954
		(Child’s age)^∧^2	–0.48	3.23	–4.16	2.40	1.00	3184	1471
		Verbs per utterance	2.19	0.25	1.74	2.72	1.00	1622	2733
		(Verbs per utterance)^∧^2	–0.59	0.09	–0.77		1.00	1744	2783
3	Age and verb/utterance ratio to predict DS marking/total medial verbs	Intercept	–4.08	0.61	–5.33	–2.91	1.00	2965	3360
		Child’s age	49.17	8.53	32.55	66.03	1.00	2931	3325
		Verbs per utterance	–0.90	0.19	–1.26	–0.54	1.00	3483	3495
4	Command/desire context to predict root medial clauses	Intercept	–2.29	0.15	–2.59	–2.01	1.00	3461	3559
		Command context	2.48	0.25	2.00	2.97	1.00	4174	4192
5	Addition of mother’s verb/utterance ratio to Model 1	Intercept	–1.81	0.06	–1.92	–1.70	1.00	7588	6279
		Child’s age	0.21	1.28	–2.27	2.86	1.00	3923	2929
		(Child’s age)^∧^2	220.95	13.01	194.69	245.92	1.00	3937	3378
		Mother’s verb/utt ratio	0.15	0.02	0.10	0.20	1.00	7087	5022

**FIGURE 9:**
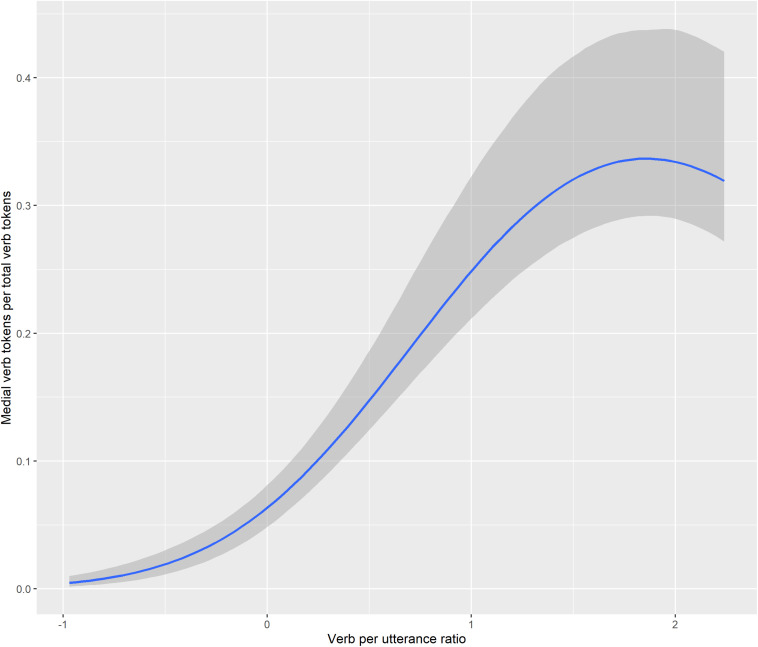
Conditional effects of verb/utterance ratio on medial verb/verb ratio.

Second, Bayesian logistic regression models were used to evaluate the relationship between children’s medial verb use and: age, verb/utterance ratio, or both age and verb/utterance ratio. The model using age and verb/utterance together was top-ranked in leave-one-out cross-validation, but with only a very small ELPD difference of -0.4 between that model and the one also including age, and a standard error of 0.5. The conditional effects of the verb/utterance ratio in this model are visualized in Figure 9, and population-level effects are given as Model 2 in Table 3.

**FIGURE 10:**
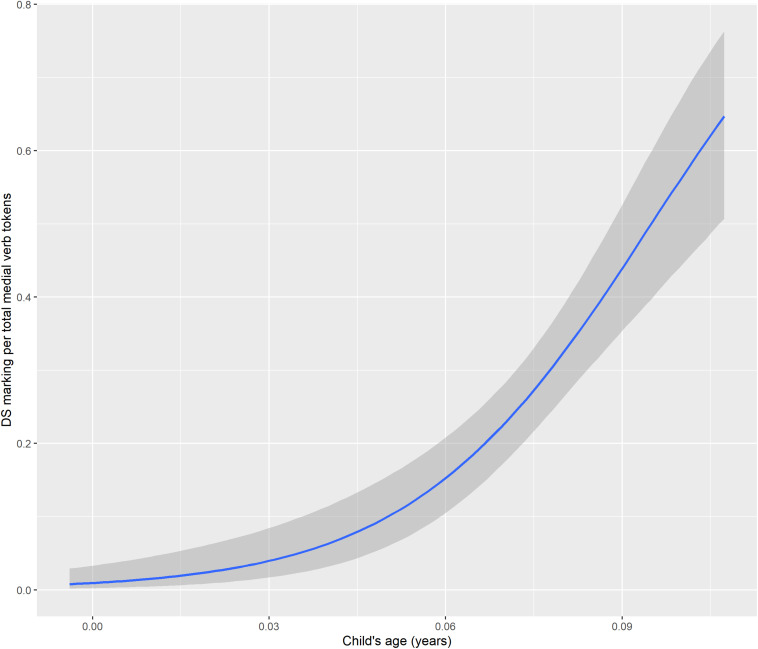
Conditional effects of age on DS marking.

Visual inspection of the children’s medial verb data yielded uncertainty around whether DS marking can be considered to increase with age. When Bayesian logistic regression models were fit to the entire dataset, including aspectual clause chains, a model predicting DS marking using a combination of age and verb/utterance ratio outperformed a null model and models using verb/utterance or age alone. Population-level effects for the best-fitting model are under Model 3 in Table 3, and the conditional effects of the child’s age on DS marking are in Figure 10.

**FIGURE 11:**
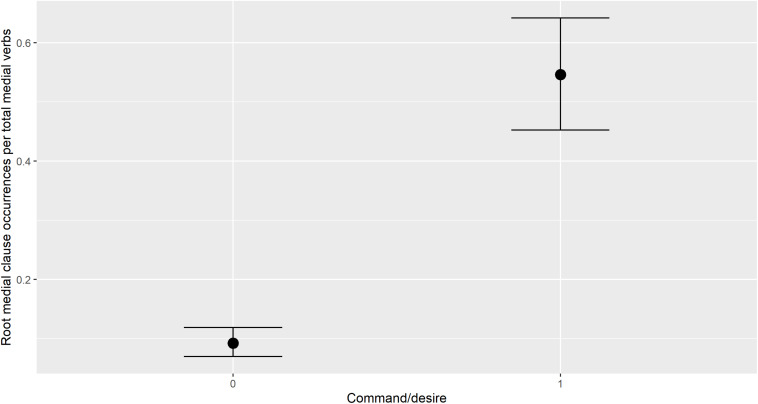
Conditional effects of command/desire context on root medial clause occurrence.

Bayesian logistic regression modeling further confirmed that the children used ‘root medial’ clauses primarily to express commands and wishes. Sarvasy (2015a) described this as one of the functions of root medial clauses in adult speech, but did not quantify it. A model in which a ‘command/desire’ context was a factor in whether a given medial verb occurred in a root medial clause far outperformed the comparison null model, in which no factors predicted root medial clause occurrence, when evaluated with leave-one-out cross-validation. Population effects for the better-fitting model are under Model 4 in Table 3, and its conditional effects are visualized in Figure 11, where the value 0 on the *x*-axis represents a non-command/desire context, and the value 1 represents a command/desire context.

**FIGURE 12:**
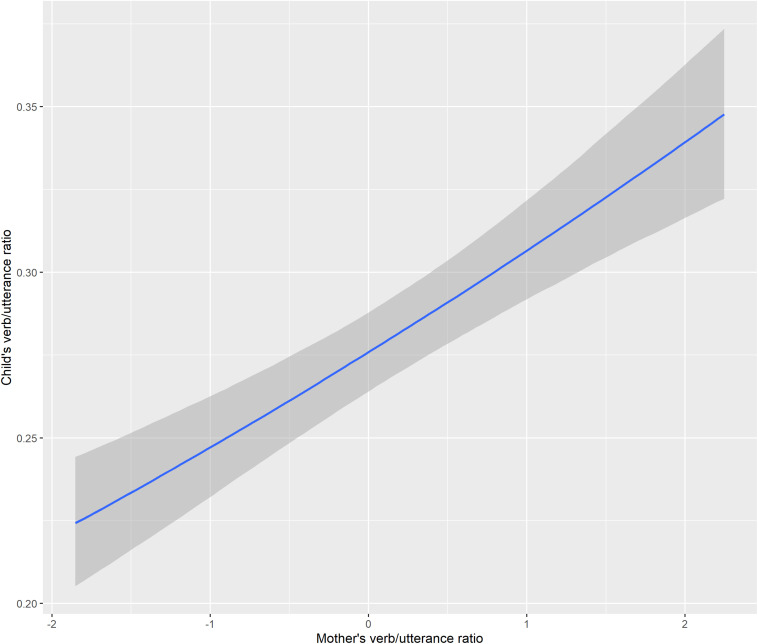
Conditional effects of mother’s verb/utterance ratio on child’s verb/utterance ratio.

The last area investigated statistically involves potential correlations between child and adult productions in a given transcript. To Model 1, I added ‘mother’s verb/utterance ratio’ as an additional factor in predicting child’s verb/utterance ratio. Indeed, as mother’s verb/utterance ratio increases, child verb/utterance ratio also increases; population-level effects are as Model 5 in Table 3, and the conditional effects are visualized in Figure 12.

To Model 2, I added ‘mother’s medial verbs/total verbs ratio’ as an additional factor in predicting child’s medial verb/total verbs ratio. The mother’s medial verbs/total verbs ratio yielded an estimated effect of lowering the child’s ratio by −0.10, but with a large amount of uncertainty (the 95% confidence intervals extend from −0.24 to 0.05). Thus, mother’s medial verb/total verb ratio is not predictive of child’s medial verb/verb ratio under this model.

## Discussion

This has been the first study of the development of clause chaining in child speech in the Nungon language of Papua New Guinea. Clause chain formation requires children to produce one or more ‘medial’ clauses ending in medial verb forms, followed by a single ‘final’ clause with a final verb that is marked for tense, mood, and subject person/number. Children’s first medial verb productions are attested at 2;4 (Abraham and TO). For both children for whom there is data at this age, most or all of the earliest medial verb tokens occur in ‘root medial’ clauses (Sarvasy, 2019b): independent uses of individual medial clauses, without any following final clause. ‘Root medial’ clauses occur more consistently than two-clause chains in the speech of TO through 2;11, when two-clause chain use begins to increase and the child seems to demonstrate more power over two-clause chain structure and semantics than in the preceding several months.

That said, one of the earliest two-clause chains produced by TO (at 2;5) is a non-conventionalized combination of verbs, with other material interpolated within the chain (example 14). This implies that TO may already have productive command of clause chain structure at this early stage, and it could reflect the limitations of the relatively sparse (1 h monthly) sampling method that TO does not demonstrate this in the five subsequent months’ recording sessions. (On the other hand, the thin sampling method could also make this chain seem more creative than it really is; a denser corpus could reveal more rote-learned patterns in child productions.)

Nungon medial verbs are obligatorily marked for switch-reference: whether the subject of the following clause will be co-referential with the present clause’s subject. Although DS-marked medial verbs are attested from early on (2;5 for TO), these only occur in root medial clauses then and for the next 5 months (through 2;10). That is, the medial verbs in TO’s first five two-clause chain productions (the total attested for her in the recording sessions between 2;4 and 2;10) are all SS. Her first DS medial verbs in two-clause chains are attested at 2;11. This implies that there could be a development-related delay in production of two-clause chains with different subjects for the two clauses: the child shows mastery of the DS-marked medial verb form in root medial contexts 6 months before she ever produces it within a two-clause chain. We await data from the new, denser longitudinal study to confirm whether the additional three children show a similar pattern.

Nungon discourse differs from that in Ku Waru of Papua New Guinea (Rumsey et al., 2020) in that DS marking is well-represented in adult speech, whether child-directed or adult-directed. In Ku Waru as in Nungon, medial verbs must indicate whether the subject of the following clause will differ from that of the present clause. But Rumsey et al. (2020) state that Ku Waru adult discourse shows a strong dispreference for DS clause chains, and this is also reflected in the makeup of child-directed speech. Accordingly, only one of the children in Rumsey et al.’s (2020) study of early Ku Waru clause chain productions ever produces a DS clause chain, and this production occurs at 4;7, over 2 years after the child began producing SS clause chains.

In Nungon, the most conventionalized two-clause chains are arguably those that verge on monoclausal constructions: the two aspectual uses of SS two-clause chains. With these constructions, other linguistic material cannot intervene between the first medial verb and the second verb, which must be a particular lexical verb. For TO, who is the only child for whom data was available in the period of 2;5–2;10, there is no indication that clause chain production begins with just aspectual clause chains, then expands to include chains describing sequential actions. TO’s three earliest spontaneous two-clause chain productions include one wholly non-conventionalized clause chain, one Continuous aspect construction, and one clause chain using the common pairing of ‘taking it up’ and ‘go.’ Further, TO’s medial verb types counts (Table 1) show no indication that she relies on a very small number of lexical verbs for her chains. Findings here therefore diverge slightly from those for Ku Waru by Rumsey et al. (2020) in that Nungon-speaking children’s earliest clause chains are a mix of more and less conventionalized lexical combinations, and there is no indication for Nungon that early chains should be analyzed as involving a single action, rather than two (If this were the case, findings for Nungon clause chain development could be compared to those for early clausal subordination in languages like English and German: Diessel, 2004; Brandt et al., 2008).

Two elements of the Nungon data point to developmental constraints on the production of complex sentences by these children: first, the relatively infrequent productions of clause chains until around the age of 3;0 (for TO, at least), after which there is marked expansion in frequency and variety of clause chains, and second, the restriction in clause chain length to just two clauses per chain until age 3;1 (for both TO and Niumen). The marked increase in frequency and variety of clause chains about 7 months after the first clause chain productions suggests that complex sentence production is at least partially constrained by development: that a ‘simple sentence’ stage does indeed precede a ‘complex sentence’ stage (as posited early by Bowerman, 1979, and as maintained by constructivist accounts such as Diessel and Tomasello, 2000, but *contra* Lust et al., 2009). Research into child acquisition of complex sentences has rarely examined the development of sentences comprising more than two clauses. The Nungon data here accords with the Ku Waru data in Rumsey et al. (2020), in that children acquiring these two Papuan languages clearly produce only two-clause chains for a period before beginning to produce chains of more than two clauses. Thus, at least for clause chains in these two languages, children can be said to go through a ‘two-clause stage,’ after which their clause chains expand in length to three clauses (for the Ku Waru children and TO), or more (for Niumen, whose two-clause stage is followed by a stage in which he produces chains of 3–5 clauses).

Another facet of Nungon clause chain production that was hypothesized to be problematic from a cognitive perspective, however, proved not necessarily so. Obligatory switch-reference marking in Nungon clause chains would seem to entail that proficient speakers must plan their clause chains at least two clauses at a time—which should be highly cognitively demanding. There are at least three potential ways to ameliorate this for child speakers. One option is that children opt not to produce clause chains at all until they are developmentally able to perform this advance planning, since subordinated or coordinated final clauses do not need to be marked for switch-reference. A second option is that children avoid producing clause chains in which the subject changes from clause to clause. They could then begin each chain with the assumption that the subject of the first clause will be maintained throughout the following clauses in the chain, and apply SS marking to each medial verb by default even if they have not planned as far as the following clause (This could be the case for children acquiring Ku Waru, though the pattern there could alternatively be attributed solely to frequencies in adult speech: Rumsey et al., 2020). A third option for dealing with the cognitive demands of switch-reference marking is that children produce clause chains that can involve changes in subjects across clauses, but use morphologically simplified medial verbs that do not force the children to indicate in advance whether the upcoming subject will differ.

The first and third option here clearly do not apply to children learning Nungon: the earliest two-clause combinations produced by Abraham and TO are clause chains, not other complex sentence types, and there is absolutely no indication that children alter medial verb forms to avoid switch-reference marking. It may be the case that children acquiring Nungon pursue the second option, preferring SS two-clause chains over DS two-clause chains from the earliest two-clause chains at 2;4–2;5 through about 2;10. But the few clause chain tokens from a single child in this critical time period mean that it is as yet unclear whether the semblance of this strategy is due to sampling. Ongoing work on the new, denser longitudinal study targeting verb productions in this age range should help to finalize this component of the analysis.

Finally, comparison of children’s early clause chain productions with their early subordinate and symmetrical coordinate sentences suggests that these three complex sentence types are all governed by the same developmental constraints on complex sentence production, but that once development has reached an adequate stage, language-specific characteristics determine distributions of the three types in a child’s speech. The very first two-clause sentences produced by the children are clause chains, not the other two types of complex sentence, but these are produced (by TO) in very low frequencies for the first 6 months (2;5–2;10). At the same age at which clause chain use by TO and Niumen begins to increase sharply, their use of the other two complex sentence types also increases, but at much lower levels.

Figures 6, 7 can be seen as support from child language data for a typological distinction between ‘clause chaining languages’ and ‘non-clause chaining languages’ (a direction of influence across linguistic sub-disciplines that is rarely achieved: Slobin and Bowerman, 2007). It is intuitively clear to language learners and descriptive linguists that some languages employ clause chaining widely and others do not, but this is difficult to quantify. For instance, the asymmetrical coordinated relationship between clauses in a clause chain can be likened to the relationship between adverbial and main clauses in languages like English (Bickel, 2010). But if an English speaker attempted to produce a clause chain-type sentence in English, using a series of clauses with gerunds instead of finite verbs, the result would be highly unnatural (as seen in the English translations of the Nungon examples in this paper), in contrast to Nungon, where the most natural translation of an English narrative is likely a series of clause chains. One way of establishing a criterial demarcation between ‘clause chaining languages’ and ‘non-clause chaining languages’ would be to require that a ‘clause chaining language’ permit chains of over three or four gerundial clauses—but this is still possible to approximate in English, albeit unnatural and decidedly rare in actual discourse. Without relying on naturalness judgments or arbitrary length limits, the child production data here shows that the Nungon language privileges clause chains over sentences with coordinated and subordinated final clauses, at least in the early stages of complex sentence production. That is, clause chains are more than two times more frequent in Nungon child speech than the other two complex sentence types. It remains to be seen whether this pattern will be replicated for children learning other ‘clause chaining languages.’

## Data Availability Statement

The datasets generated for this study are available on request to the corresponding author.

## Ethics Statement

The studies involving human participants were reviewed and approved by Australian National University Human Ethics Committee. Written informed consent to participate in this study was provided by the participants’ legal guardian/next of kin. Written informed consent was obtained from the individual(s), and minor(s)’ legal guardian/next of kin, for the publication of any potentially identifiable images or data included in this article.

## Author Contributions

The author confirms being the sole contributor of this work and has approved it for publication.

## Conflict of Interest

The authors declare that the research was conducted in the absence of any commercial or financial relationships that could be construed as a potential conflict of interest.

## Abbreviations

1, 2, 3: 1st, 2nd, 3rd person
ADV: adverbializer
ASSOC: associate plural
ATT: attention marker
BEN: benefactive
BT: baby talk form
COMIT: comitative
CONJ: conjunction
DEP: dependent
DIST: distal
DS: different subject
DU: dual
EMPH: emphatic
FOC: focus
IMP: immediate imperative
INFR: inferred imperfective
INTJ: interjection
LOC: locative
MV: medial verb
NEAR: near distance
NEG: negator
NF: near future
NMZ: nominalizer
NP: near past
O: object
PL: plural
POSS: possessive
PRES: present
PRO: pronoun
PROX: proximal
RED: reduplicated
RF: remote future
RP: remote past
SEMBL: semblance
SG: singular
SPEC: specifier
SR: switch-reference
SS: same subject
SUB: subordinator
TOP: topic
UNINT: unintelligible.
